# Reconstruction of Avian Reovirus History and Dispersal Patterns: A Phylodynamic Study

**DOI:** 10.3390/v16050796

**Published:** 2024-05-16

**Authors:** Giovanni Franzo, Claudia Maria Tucciarone, Giulia Faustini, Francesca Poletto, Riccardo Baston, Mattia Cecchinato, Matteo Legnardi

**Affiliations:** Department of Animal Medicine, Production and Health (MAPS), University of Padua, 35020 Legnaro, Italy; claudiamaria.tucciarone@unipd.it (C.M.T.); giulia.faustini.1@phd.unipd.it (G.F.); francesca.poletto.1@studenti.unipd.it (F.P.); riccardo.baston@phd.unipd.it (R.B.); mattia.cecchinato@unipd.it (M.C.); matteo.legnardi@unipd.it (M.L.)

**Keywords:** ARV, phylodynamic, evolution, vaccination, selection

## Abstract

Avian reovirus (ARV) infection can cause significant losses to the poultry industry. Disease control has traditionally been attempted mainly through vaccination. However, the increase in clinical outbreaks in the last decades demonstrated the poor effectiveness of current vaccination approaches. The present study reconstructs the evolution and molecular epidemiology of different ARV genotypes using a phylodynamic approach, benefiting from a collection of more than one thousand sigma C (σC) sequences sampled over time at a worldwide level. ARVs’ origin was estimated to occur several centuries ago, largely predating the first clinical reports. The origins of all genotypes were inferred at least one century ago, and their emergence and rise reflect the intensification of the poultry industry. The introduction of vaccinations had only limited and transitory effects on viral circulation and further expansion was observed, particularly after the 1990s, likely because of the limited immunity and the suboptimal and patchy vaccination application. In parallel, strong selective pressures acted with different strengths and directionalities among genotypes, leading to the emergence of new variants. While preventing the spread of new variants with different phenotypic features would be pivotal, a phylogeographic analysis revealed an intricate network of viral migrations occurring even over long distances and reflecting well-established socio-economic relationships.

## 1. Introduction

Avian reoviruses (ARVs), classified under the species *Avian orthoreovirus*, within the genus *Orthoreovirus*, and belonging to the family *Reoviridae* (https://ictv.global/taxonomy; accessed 21 March 2024), are non-enveloped double-stranded RNA viruses. The viral genome comprises 10 segments, which are distinguished by size through electrophoretic mobility. These segments are categorized into three classes: the L class (L1–L3), M class (M1–M3), and S class (S1–S4) [1,2]. Except for S1, which encodes three proteins (i.e., p10, p17, and σC), all segments are monocistronic [1]. ARV is associated with various poultry diseases that, despite their generally low mortality, lead to significant morbidity and economic losses [3]. The primary clinical syndrome is viral arthritis and tenosynovitis in poultry and turkeys. Although less established, its connection to other clinical conditions such as runting–stunting syndrome, hepatitis, myocarditis, hydropericardium, malabsorption syndrome, and disorders of the respiratory, enteric, and central nervous systems, has been noted [4]. ARV infection is also linked to immunosuppression [5], likely due to lymphoid depletion in immune system organs and the induction of cell apoptosis [6,7,8]. When layer and breeder flocks are affected, decreases in egg production, fertility, and hatchability are noted, along with vertical transmission to offspring [4].

Not all reoviruses are pathogenic, and their virulence can vary greatly among isolates, which may differ in the timing and severity of clinical outcomes and transmission potential [9]. Over recent decades, there has been a significant increase in the number of reported and diagnosed cases in several countries [10,11]. Concurrently, the understanding of the genetic variability of ARV has expanded. The σC protein, which forms the homotrimer responsible for viral attachment, is also targeted by neutralizing antibodies [2,12]. Owing to its biological implications and greater variability compared to other genome regions, the S1 segment is commonly used for sequencing, phylogenetic analysis, and molecular epidemiology studies. To date, seven genetically distinct genotypes have been identified based on the analysis of this region [10,13,14,15].

Vaccination remains a key strategy for control, and both commercial inactivated and attenuated vaccines have been available since the early 1980s [11]. However, except one inactivated vaccine, all registered vaccines are based on a limited number of genotype 1 strains [16]. There is consistent evidence of a lack of protection against field strains even among vaccinated animals [17,18]. Since σC induces neutralizing antibodies, the variability in this protein contributes to the lack of protection. In an effort to control disease, the use of inactivated autogenous vaccines is almost universal in some countries, including the US. However, this approach presents challenges, including difficulties in identifying the circulating strain and timely production, as well as the impossibility of achieving proper priming in the absence of an effective live attenuated vaccine [4,11]. Moreover, autogenous vaccines are not permitted in several countries [10].

For these reasons, a thorough understanding of ARVs’ molecular epidemiology, strain and genotype distribution, and migration patterns would be valuable for planning and optimizing future control measures (e.g., new vaccines) and for attempting to limit the introduction and mixing of heterogeneous strains from different origins that further complicates infection control due to limited cross-protection among distantly related strains. Conversely, while the global circulation of ARV is recognized, no systematic study has been conducted to describe the overall situation and identify the underlying determinants. In the present study, a global collection of complete or nearly complete ARV S1 sequences has been analyzed using a phylogenetic and phylogeographic approach to address this knowledge gap.

## 2. Materials and Methods

### 2.1. Sequence Dataset Preparation

Available ARV σC coding sequences were downloaded from Genbank and aligned with MAFFT v7 [19] together with the reference dataset provided by Lu et al. [13]. Only sequences whose collection date and country were available and with adequate quality (i.e., absence of obvious misalignment, unknown bases, premature stop-codons or frameshift mutations) were maintained in the dataset. A preliminary tree was reconstructed using IQ-Tree v2.3.2 [20] selecting the substitution model with the lowest Bayesian Information Criteria (BIC) calculated using the same software. All strains clustering with vaccine strains were identified and removed from the study. Recombination occurrence was assessed using GARD v0.1 [21] and the strength of the phylogenetic signal was assessed through likelihood mapping analysis implemented in IQ-Tree [17], while the temporal signal was investigated using TempEST v1.5.3 [19].To perform a phylodynamic analysis on ARV, the complete dataset was down-sampled by randomly selecting a maximum of four strains for each country–year pair. This approach was useful in reducing the computational burden and enhancing the following converging and mixing. At the same time, by performing repeated analysis on more balanced, but randomly generated, datasets, it was possible to compensate for and evaluate the effect of differential sequencing activity over time and space, as suggested by Layan et al. [20].

Genotype-specific datasets were also generated, and the same subsampling procedure was applied if the number of available sequences was enough to create inter-dataset variability. Otherwise, a single dataset was maintained.

### 2.2. Phylodynamic and Phylogeographic Analysis

The selected datasets were analyzed to reconstruct several population parameters, including time to the most recent common ancestor (tMRCA), substitution rate (substitutions/site/year; s/s/y), and viral population dynamics using the Bayesian serial coalescent approach implemented in BEAST 1.10 [21]. For each dataset, the nucleotide substitution model was selected based on the BIC score calculated using JmodelTest v2.1.10 [22]. The molecular clock was selected calculating the marginal likelihood estimation through path-sampling and stepping-stone methods, as suggested by Baele et al. [23]. The non-parametric Bayesian Skygrid was selected to reconstruct viral population changes over time (relative genetic diversity: Effective population size∙generation time; N_e_∙τ) [24]. For the genotype-specific datasets, a discrete state phylogeographic analysis was also performed, as described by Lemey et al. [25], implementing an asymmetric migration model with Bayesian stochastic search variable selection (BSSVS), allowing the identification of the most parsimonious description of the spreading process and calculation of a Bayesian Factor (BF) indicative of the statistical significance of the inferred migration path between geographic areas. Due to the sparse nature of the sequence–country combination and the likely missing sampling in several countries, and in order to obtain a more balanced dataset, countries were aggregated in macro-areas considering their spatial proximity and geopolitical factors (i.e., Africa, Asia, Central America, Europe, the Middle East, North America, and South America). Two independent runs of 200 million generations were performed. The log and tree files were merged using logcombiner after the removal of a burn-in of 20%. Results were analyzed using Tracer 1.7 and accepted only if the estimated sample size (ESS) was greater than 200 and the convergence and mixing were adequate. Parameter estimation was summarized in terms of mean and 95% highest posterior density (95HPD). Maximum clade credibility (MCC) trees were constructed and annotated using TreeAnnotator (BEAST 1.10 package). SpreaD3 [26] was used to calculate the BF associated with each migration route. All non-zero transition rates among countries were considered significant if the calculated BF was greater than 10. Additional summary statistics and graphical outputs were generated using homemade R scripts v4.2.2 [27].

### 2.3. Selective Pressure Analysis

The action of selective pressures was evaluated on the complete σC dataset, including all genotypes, using an approach based on the non-synonymous to synonymous substitution rate calculation (dN/dS). Briefly, a dN/dS higher, equal, or lower than 1 suggests diversifying, neutral, and purifying selection, respectively [18]. Pervasive selective pressures were analyzed using FUBAR [28] and FEL [29], while episodic selection occurrence was assessed with MEME [30], implemented in HyPhy [31]. The significance level was set at posterior probability (PP) > 0.9 and *p*-value < 0.05 for FUBAR and FEL and MEME, respectively.

The difference in selective pressure strength among genotypes was assessed using contrast-FEL [32]. This approach was developed to detect individual alignment sites where two (or more) sets of branches in a phylogenetic tree have different dN/dS ratios. The false discovery rate (FDR)-corrected q-value of 0.2 was accepted as an index of overall evidence of a site-specific difference in selective pressure, while a *p*-value < 0.05 was accepted for each genotype pair comparison.

The occurrence of directional selection, driving the viral evolution away from the vaccine, was tested on the amino acid alignment genotype 1 strains using the Fast, Unconstrained Bayesian AppRoximation for Inferring Selection (FADE) method [33], rooting the tree on the clade including the vaccine strain (background) and selecting the rest of the tree as foreground. The significance level was set at *p*-value < 0.05.

### 2.4. Homology Modelling

The sequence of a representative strain (S1133) belonging to genotype 1 was selected, translated at the amino acid level, and used as the template for homology modeling, performed with the SWISS-MODEL server [34]. The final model of the trimeric structure was plotted and edited using Chimera v 1.16 [35]. The prediction of pockets and ligand binding sites from the protein structure was performed with PrankWeb v2 [36]. Only regions with a probability higher than 10% were considered.

## 3. Results

### 3.1. Datasets

**Figure 1:**
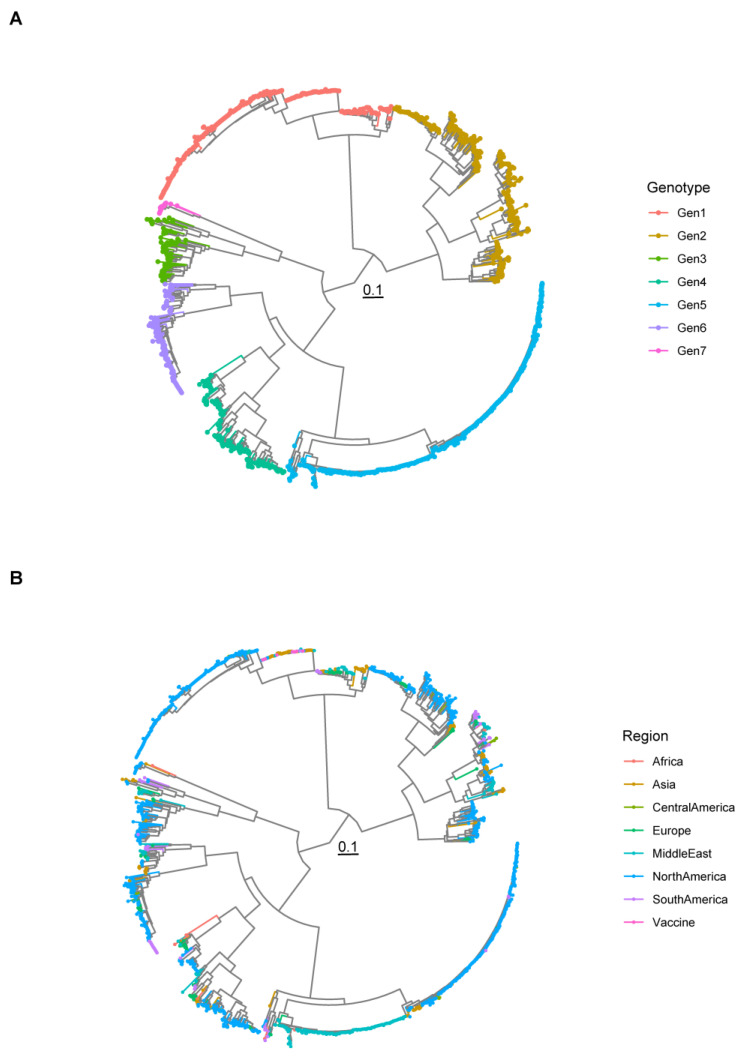
Maximum likelihood phylogenetic tree reconstructed using the σC sequences of all strains included in the study. The classifications into genotypes (**A**) and based on the region of origin (**B**) are color-coded.

A total of 1392 sequences were included in the final dataset, originating from 46 countries, seven macro-areas, and spanning a time interval from 1984 to 2023. Seven clusters, corresponding to previously defined genotypes, were identified. Strains originating from several counties were classified in the same genotype, in the absence of apparent geographical clustering (Figure 1 and Figure S1). A summary of included sequence features, classified based on genotype, is reported in Table S1.

For genotype-specific analysis, four randomly generated datasets were created for genotypes 1, 2, and 5, while, for 3, 4, and 6, the whole sequence dataset was considered. Due to the limited number of available sequences, no phylodynamic analysis was performed for genotype 7.

### 3.2. Phylodynamic and Phylogeographic Analyses

The average substitution rate of AVR was estimated 1.69 × 10^−3^ s/s/y [95HPD = 1.08 × 10^−3^–2.20 × 10^−3^], and the tMRCA was inferred in 1392.11 [95HPD = 1128.94–1601.32]. Substantially, overlapping results were obtained by subsampling the original dataset (Figure S2).

The estimated substitution rate and tMRCA for each genotype are reported in Table S1. When multiple, randomly generated datasets were analyzed, the consistency of the results was verified and the reported summary statistics were based on the combination of all run results.

**Figure 2:**
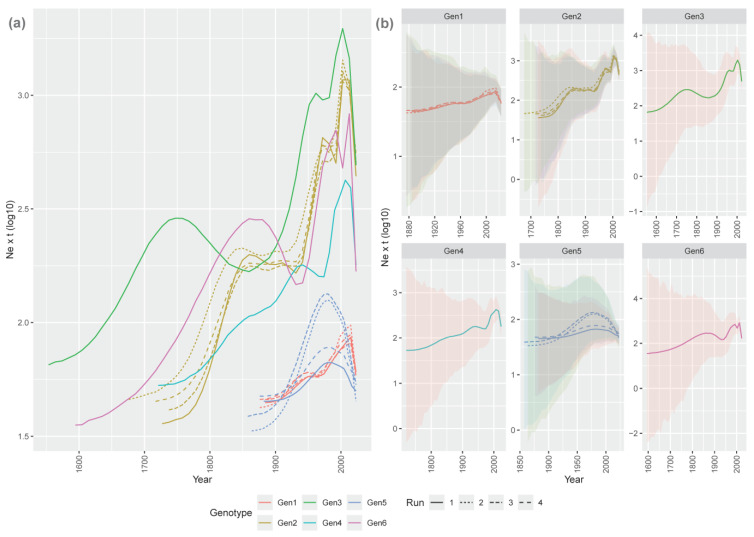
(**a**) picture. Mean relative genetic diversity (Ne × t) of the Avian reoviruses (ARVs) genotype (color-coded) population over time. The results of the independent runs are depicted with different line styles. (**b**) ARVs’ mean relative genetic diversity (Ne × t) of the ARV genotypes is reported in different panels. The 95HPD is superimposed as shaded areas.

The reconstructed viral population dynamics varied considerably among genotypes (Figure 2), although some common patterns were identified. The origin of and rise in genotype 3 were predicted to have occurred in the late XVII century, persisting until about the middle of the XVIII century, when a decline occurred. In parallel, the origin of and marked increase in genotypes 6 and 2 were subsequently observed. A stationary phase spanned the period ~1800–1900 for the above-mentioned genotypes, with the slight exception of genotype 4, showing a constant, progressive increase from its origin onward. Beginning from the 20th century, a new population expansion phase affected all genotypes and was accompanied by the emergence of and rise in genotypes 1 and 5, continuing up to the new millennium, with some significant fluctuation, and declining only in the final years of the study (Figure 2).

**Figure 3:**
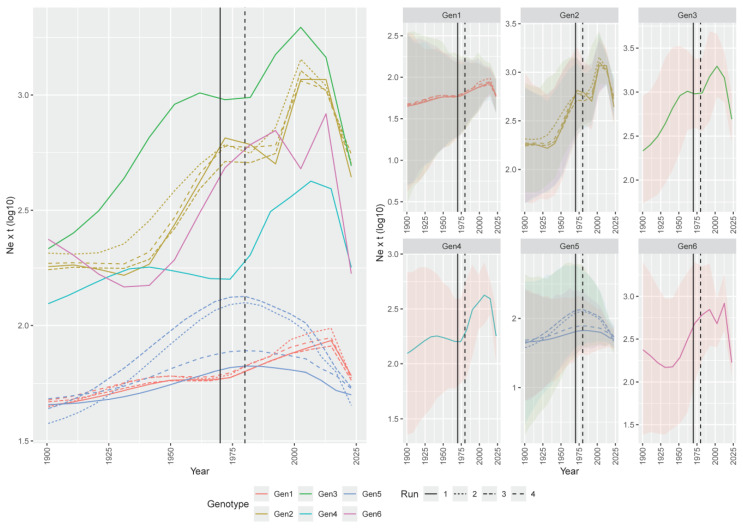
Reconstruction of the ARV population dynamics, with focus starting in the 20th century. Left picture. Mean relative genetic diversity (Ne × t) of the ARV genotype (color-coded) population over time. The results of the independent runs are depicted with different line styles. Right picture. The ARVs’ mean relative genetic diversity (Ne × t) of the ARV genotypes is reported in different panels. The 95HPD is reported as a shaded area. The period when inactivated and live vaccines were developed and commercialized is reported as solid and dashed lines.

A more dedicated focus on the XX century revealed that all genotype populations significantly increased until the 1970s, followed by a stationary or slight decline lasting about two decades, and a new rise thereafter. A new stability or minor decline affected some genotypes in the early 2000s. Genotype 5 was the main exception, showing a parabolic curve peaking at around 1980 and declining thereafter (Figure 3).

**Figure 4:**
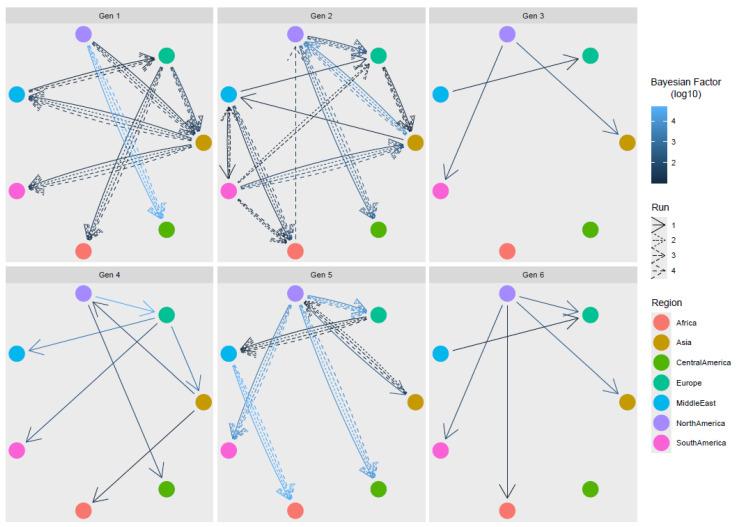
ARVs statistically supported migration rates. Different genotypes are reported in separate panels. Edges among regions (color-coded) represent statistically supported migration rates. The arrow color intensity is proportional to the BF value. The results of multiple runs are reported with different line styles.

The evaluation of statistically significant migration rates detected several connections among macro-areas, regardless of the considered genotype (Figure 4 and Figure S1). When migration events were inferred on multiple, subsampled datasets, consistent results were always achieved with the only partial exception of genotype 2, where the link between Africa and North America was inferred only once, and the link between the Middle East and South America was identified in multiple runs, but with variable directionality.

Overall, North America appeared to be the main source of viral introduction to other areas. Other regions featured more complex patterns. For example, Europe was involved in the exportation of genotypes 1, 2, and 4 to Asia, but was, at the same time, importing ARVs from other macro-areas, the Middle East having a pivotal role. Intermediate steps and circular patterns were also encountered, e.g., from Europe to Asia, from Asia to the Middle East, and from the Middle East to Europe. Strain importation to Africa seemed mediated by different sources, i.e., genotype 1 from Europe, genotypes 2 and 5 from the Middle East, and genotype 5 from Asia and from North America, while, for other regions, unilateral, exclusive importation events occurred, i.e., the directional importation of ARSs from North to Central America and, to a lesser extent, to South America, for which the contribution of other sources was also estimated.

### 3.3. Selective Pressures Analysis

**Figure 5:**
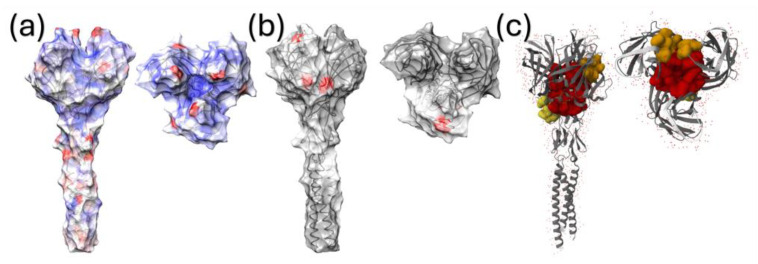
The quaternary structure of the σC protein portion, for which the experimental structural model was available, is depicted from different perspectives. The surface of figure (**a**) is colored from red (positive values) to blue (negative values) according to the estimated dN-dS value, estimated using FUBAR. In (**b**), the sites under episodic diversifying selection, estimated using MEME, are reported. (**c**) depicts the inferred pockets of the σC protein with binding activity potential.

The analysis of pervasive selective pressures performed with FUBAR and FEL detected sites under a significant diversifying selection at positions 5, 23, 24, and 27 of the σC protein. None of these sites were in the region of the protein for which the structure had been experimentally determined. However, when the difference in the synonymous to non-synonymous substitution rate was plotted on the protein surface (Figure 5a), a higher diversification tendency affected the stalk region and some sites on the head domain, especially those lining the lateral (around AA 254) and top protein regions, facing the central depression formed by the monomers (AA284). The analysis of episodic diversifying selection detected additional sites, including amino acids 13, 23, 24, 27, 28, 30, 32, 46, 56, 59, 63, 238,265, 271, 272, and 297. Four of those, i.e., 238, 271, 272, and 297, were exposed on the surface of the globular σC head (Figure 5b).

The comparison of selective force strength acting on ARV genotypes identified several sites under differential selection, (i.e., 39, 64, 125, 157, 170, 171, 175, 196, 230, 254, 255, 263, and 279) some exposed on the protein surface, both in the stalk and head. A detailed depiction of the significant pairwise comparison is reported in Figure S3.

A directional evolution, differentiating ARV strains belonging to genotype 1 from vaccine strains, was detected at positions 10 (R → K and R → I), 35 (E → D and E → K), 76 (T → I), 123 (G → E, G → S and G → I), 205 (R → K), and 230 (M → I). Potential protein binding sites were estimated in regions located in the central part and borders of the pocket formed by the three σC monomers, and at the base of the globular head (Figure 5c)

## 4. Discussion

Avian reovirus infection has been recognized for decades and causes significant losses for the poultry industry that have long been effectively controlled by vaccination [4,37]. However, ARVs’ detection and related diseases have increasingly been reported in recent years [8,9]. The incomplete cross-protection provided by available vaccines against newly described variants has been identified and demonstrated as a pivotal cause [15,38,39]. However, the epidemiological and evolutionary patterns have not been investigated from a global perspective, and the underlying causes have never been investigated. This study demonstrates the ancient origin of ARVs, well predating the first clinical description, as is common for several animal infections. Interestingly, the origins of all genotypes, including those identified more recently, were estimated at least a century ago. This result is not unexpected, since the development and widespread application of molecular techniques have only been routinely applied in the last decades, leading to recent improvements in ARV molecular epidemiology. The actual sequencing of archive samples collected since 1984 and their classification into genotypes different from the considered “historical” genotype 1 support the premise of the longstanding circulation of multiple, distantly related variants [40]. On the contrary, genotypes 2, 3, 4, and 6 may have emerged before the divergence of genotype 1 at the beginning of the 20th century, although the presence of quite broad 95% HPD intervals in tMRCA estimations must be stressed. Moreover, a potential underestimation of the origin of genotype 1 due to the removal of vaccine-related strains cannot be excluded.

An analysis of viral population dynamics provides further insight into the relationships among genotypes and their interaction with the poultry production system, although Skygrid population curves cannot be considered as a strict proxy for viral prevalence, and thus their magnitude cannot be compared among genotypes (i.e., higher relative genetic diversity values of one genotype do not imply its higher prevalence) [41]. However, temporal trends can still be considered and compared. Genotype 3 was the first to emerge, and steeply increased until about the XVII–XVIII century, when a decrease was observed in parallel to the emergence of and rise in genotypes 6, 2, and 4. The competition for a limited host population, either direct or mediated by at least partial cross-protection, can be suggested. The subsequent emergence of two additional genotypes (1 and 5) and the increase in population size of all ARV groups occurred in the 20th century, likely linked to the progressive development of modern poultry farming. Increased host populations, higher animal densities, the presence of stress factors, and immunosuppressive agents created favorable conditions for ARV circulation and population increase [42,43,44].

Interestingly, a slowdown was observed for some decades around the 1980s. During the 1970s and 1980s, inactivated and live vaccines based on genotype 1 strains were initially developed and commercialized [9], and their increasing application may have effectively counteracted ARV circulation. However, despite an overall common pattern identified among genotypes, some variations in viral population constraining were observed, and, regardless of the genotype, the efficacy appeared transient, since a new significant upsurge in viral effective population size occurred in the 1990s/2000s. The observed trend seems to contrast with the evidence of more severe clinical outbreaks occurring only after 2010 [8,9]. Moreover, because of the use of genotype 1-based vaccines, a more marked decrease in this subgroup would have been expected, which was not the case.

Firstly, vaccines may provide non-sterilizing immunity, protecting from severe clinical signs but still allowing animal infection and excretion [45,46,47,48,49]. Therefore, the reduction in viral circulation due to higher population immunity could have been balanced by an increased host population size and broader animal trade and mixing. In this sense, it must be considered that vaccination was not universally applied, and improper vaccination approaches were common, leaving some pockets for viral persistence and expansion, as proven for other poultry pathogens [50]. Moreover, the sequencing of strains from countries previously not sampled could have also “artificially” increased the viral population size estimations in more recent times. The current emergence of clinical signs could thus be determined by reaching a “high infectious pressure threshold”, as suggested for infectious bronchitis virus (IBV) [51], acting in concert with other managerial practices or infectious agents.

An alternative but not conflicting hypothesis can involve viral evolution in a partially immune environment, leading to a progressive vaccine escape. Cross-protection, although limited, can occur among genotypes, as suggested by experimental evidence [15] and by the apparent competition among genotypes in the “pre-vaccination” era. Several sites located on the σC surface were proven to be under diversifying selection, as expected for one of the main targets of the host immune response [4,39], including the vaccine-derived one. Several of these sites were located in protein pockets and binding sites, likely targets of neutralizing antibodies [52]. In particular, most of these sites were under episodic diversifying selection, which testifies to the selective bursts that might occur and act for a limited period (e.g., after vaccine introduction, emergence of competing strains, introduction in new regions, etc.) [30], rather than a constant pressure enduring the entire ARV history. Accordingly, forces with different strengths were detected among genotypes acting on several sites of the σC, which might reflect a differential interaction with immune-induced pressures. Unfortunately, a proper understanding of the forces acting differentially on specific sites is challenging, since several contributing factors might be involved, ranging from the differential cross-reactivity of different protein regions against antibodies induced by commercial vaccines to the occurrence of compensatory mutations, or interactions with different environments. The diverse patterns might thus represent different evolutionary strategies adopted over time to counteract the new immune environment.

The lack of a more marked effect of vaccination against genotype 1 compared to the others was less expected. Also, in this case, several explanations can be provided. Some (e.g., absence of complete vaccine coverage, non-sterilizing immunity) have already been mentioned. Additionally, it must be stressed that the reconstruction of genotype 1 population dynamics was performed after the exclusion of vaccine-like strains. In fact, including sequences derived from non-evolving strains in an evolutionary analysis would have led to biased and incorrect results [19]. However, confidently differentiating vaccine strains from vaccine-like strains was impossible, and a full removal was necessary. Only strains belonging to the same genotype but distantly related to vaccines were thus analyzed. Partial cross-protection against these strains can be hypothesized, leading to a lower-than-expected reduction in viral population size. The occurrence of biologically significant differences among different strains of the same genotypes was proven, and features of other proteins in addition to σC could also be involved [7]. A within-genotype progressive evolution to escape from vaccine immunity is also likely and has been confirmed by the occurrence of several sites under significant directional selection, displaying amino acid mutations differentiating them from the phenotypic features of vaccine and vaccine-like strains. Finally, the development and application of autogenous vaccines against all genotypes likely confounded the overall scenario, posing patchy and variable selective constraints.

Based on these results, infection, viral circulation, and, with all evidence, clinical disease control merely through vaccination is ineffective due to the currently available vaccines’ weaknesses, but also to viral plasticity. Besides acting on vaccination strategies, more effective biosecurity measures should be considered to limit viral circulation and infectious pressure. Support for this statement came, again, from an inspection of viral population dynamics, where a decline, or at least a steady phase, was observed for all genotypes around 2000–2010. The major influenza outbreaks occurring in several regions in that period forced the implementation of stricter monitoring, surveillance, and biosecurity measures that might also have indirectly, but effectively, acted against ARVs, similarly to what was suggested for IBV in Asia [50].

Despite the relevance of limitations to viral spreading, the analysis of overall ARV dispersal patterns suggests unconstrained viral circulation, even among distantly related regions, with genetically related strains dispersed globally and multiple genotypes circulating in the same area. Due to the limited and biased sequence availability, and the longstanding viral circulation, the precise reconstruction of ARV circulation over time was beyond the scope of this work. Nevertheless, the significant contacts among regions and the dominant viral flows could still be identified, testifying to a complex network involving different genotypes. While North America was the main nucleus of viral dispersal, other significant contacts among areas were reported and these reflected previously described patterns for other avian infectious diseases and/or mirrored well-established economic and commercial relationships among areas. The bidirectional flow of viral strains involving Asia and Europe was already reported for IBV [53,54], as well as the “mediator role” played by the Middle East in this migratory path [55]. Similarly, different areas were involved in ARVs’ introduction to Africa, reflecting the changing economic relationships and interests of different areas of the world with different countries of this continent. Conversely, the strong influence of North America justifies its primary role in the importation of several ARV genotypes into Central America. Without overestimating the results of the phylogeographic analysis, the massive ARV worldwide dispersal appears clear, following the same pathways shaping the epidemiology of other impacting poultry viruses. Because of the relevance of ARV diversity in its control, especially ascribable to the absence of broadly effective vaccines and the need for producing autogenous vaccines, further efforts should be made to identify, monitor, and prevent viral circulation, which should also have obvious, indirect effects on other pathogens’ control as well. This study is not free of limitations, the main one being the scarce and sparse sequence availability. Additionally, the absence of proper metadata prevented the inclusion of several countries or time periods for which sequence data were available. Therefore, researchers should be encouraged to provide as much information as possible to properly characterize sequenced strains and thus contribute to the understanding of the epidemiology and evolution of infectious diseases. Nevertheless, the consistency of the results obtained through multiple runs supports their reliability, at least when the overall trends were considered [20,56].

While the present study contributed to the understanding of Reovirus epidemiology and evolution, it also highlights the limitations of current control strategies. Further work should be performed to improve the available tools, with a particular focus on new vaccine development. Therefore, the genetic data considered here should be linked to phenotypic and structural data, and in silico analyses should be validated with appropriate experimental trials.

## 5. Conclusions

The present study demonstrates the ancient and progressively increasing unconstrained worldwide circulation of ARVs, involving all genotypes, in spite of vaccine development and application. While understanding the reasons behind the recent increase in clinical outbreaks is challenging, a combination of poor vaccine efficacy, intensification of farming systems, and continuous viral evolution is likely involved. Therefore, efforts should be made to constrain viral circulation more effectively with improved monitoring and biosecurity measures, limiting the co-occurrence of different strains with variable immunological features in the same area, decreasing infectious pressure, and hampering the viral evolution potential.

## Data Availability

All sequences were obtained from freely accessible datasets (https://www.ncbi.nlm.nih.gov/nucleotide/; accessed 16 January 2024).

## Supplementary Materials

The following supporting information can be downloaded at https://www.mdpi.com/article/10.3390/v16050796/s1, Figure S1. Estimated geographical distribution of different genotypes and ARVs over time. Different macro-areas have been color-coded. When multiple runs were performed, they were depicted as different rows. Figure S2. Boxplot depicting the tMRCA posterior probability (upper panel) and mean evolutionary rate (expressed in base-10 logarithm) (lower panel). Graphs are depicted for the genotype for which multiple runs were performed. Figure S3. Graphical summary of the amino acid positions where a differential diversifying selection was detected among genotypes. The inner circle reports the involved position, while, in the outer one, the involved genotype pairs are reported. The size of the slices is proportional to the dN/dS difference (reported within the slice) for each genotype pair. Table S1. Summary of the metadata of sequences included in the study. For each genotype, the macro-area, country, and collection period are reported. Times to most recent common ancestor and evolutionary rate are also reported.
