# Supplementary material for: Reconstruction of Avian Reovirus History and Dispersal Patterns: A Phylodynamic Study
Source: Viruses. 2024 May 16;16(5):796. doi: 10.3390/v16050796 (PMC11125613; doi:10.3390/v16050796)
Supplement: Supplementary file 1 [file viruses-16-00796-s001.zip › viruses-2960796-supplementary.pdf]

Table S1. Summary of the metadata of sequences included in the study. For each genotype, the macroarea, country, and collection period are reported. Time to most recent common ancestor and evolutionary rate are also reported.

Genotype	Region (number of sequences)	Countries	Date range	tMRCA [95HPD]	substitution rate (s/s/y) [95HPD]
Genotype 1	Africa (4)	Egypt	1984–2023	112.55 [79.65–148.35]	2.31×10^{-3} $[1.82\times10^{-3}–2.83\times10^{-3}]$
	Asia (69)	Bangladesh, China, India, Indonesia, Malaysia, Vietnam			
	Central America (4)	Costa Rica, Mexico			
	Europe (12)	Germany, Hungary, Poland, Romania, Russia			
	Middle East (25)	Iran, Israel, Oman			
	North America (176)	Canada, USA			
	South America (9)	Brazile, Chile			
Genotype 2	Africa (4)	Egypt, Ghana, Morocco	2005–2023	237.47 [177.61–300.45]	1.80×10^{-3} $[1.418^{-}\times10^{3}–2.21\times10^{-3}]$
	Asia (31)	Bangladesh, China, Indonesia, Malaysia, Philippines, South Korea			
	Central America (4)	Costa Rica, Nicaragua, Panama			
	Europe (24)	Bulgaria, Hungary, Poland, Romania, Russia, Spain			
	Middle East (30)	Iran, Israel, Turkey, United Arab Emirates			
	North America (191)	Canada, USA			
	South America (12)	Argentina, Brazil, Chile, Colombia			
Genotype 3	Africa (2)	Egypt	2007–2021	381.56 [290.61-469.99]	1.22×10^{-3} $[8.88\times10^{-4}–1.56\times10^{-3}]$
	Asia (8)	China, Malaysia			
	Europe (5)	Hungary, Romania, Russia			
	Middle East (4)	Iran, Israel			
	North America (53)	Canada, USA			
	South America (4)	Bolivia, Chile, Colombia, Jamaica			
Genotype 4	Africa (7)	Egypt, Morocco, South Africa	1984–2023	226.83 [150.01–307.91]	2.03×10^{-3} $[1.51\times10^{-3}, 2.55\times10^{-3}]$
	Asia (9)	China, Japan, Malaysia, Philippines			
	Central America (1)	Nicaragua			

	Europe (26)	France, Germany, Greece, Hungary, Netherlands, Poland, Romania, Russia			
	Middle East (5)	Israel, Lebanon, Oman, United Arab Emirates			
	North America (92)	Canada, USA			
	South America (4)	Argentina, Brazil, Colombia			
Genotype 5	Africa (25)	Cote d'Ivoire, Egypt	1984–2022	101.55 [60.70–147.82]	2.45^{-3} [1.74×10^{-3}–3.27×10^{-3}]
	Asia (21)	China, Japan			
	Central America (5)	Costa Rica, Nicaragua			
	Europe (6)	Germany, Switzerland, Ukraine			
	Middle East (132)	Israel, Saudi Arabia			
	North America (231)	Canada, USA			
	South America (9)	Brazil, Chile, Colombia			
Genotype 6	Africa (1)	Egypt	2004–2022	284.27 [167.55–430.47]	1.33×10^{-3} [9.24×10^{-4}–1.77×10^{-3}]
	Asia (11)	China, Japan, Malaysia, Philippines			
	Central America (3)	Mexico, Panama			
	Europe (14)	Bulgaria, Greece, Hungary, Romania, Russia, Spain, Ukraine			
	Middle East (8)	Iran, Israel, Saudi Arabia, Turkey			
	North America (67)	Canada, USA			
	South America (21)	Brazil, Chile, Colombia			
Genotype 7	Africa (1)	Cameroon	2013–2019	Not calculated	Not calculated
	Asia (1)	Viet Nam			
	North America (14)	Canada, USA			

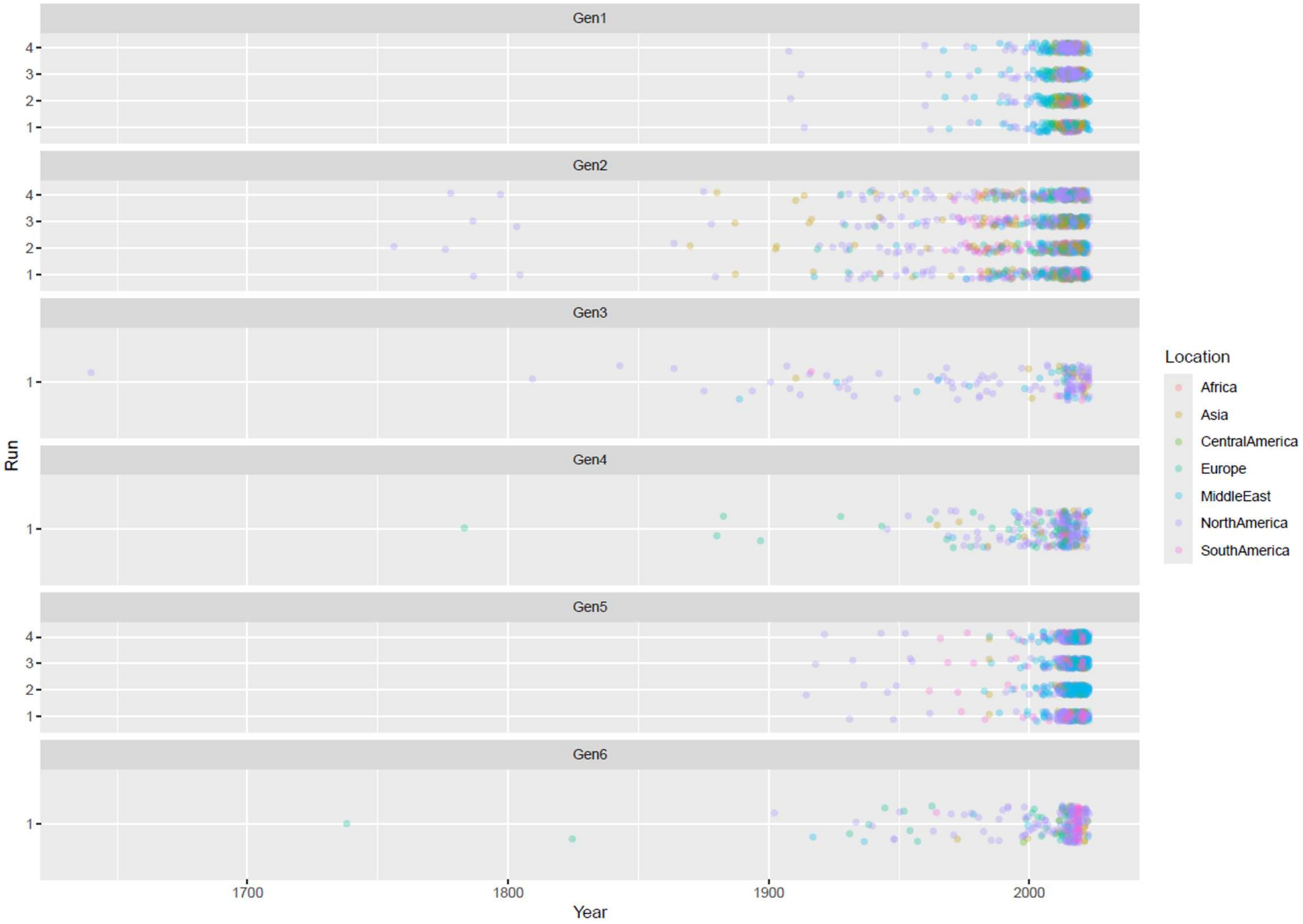

Figure S1. Estimated geographical distribution of different genotypes and ARVs over time. Different macro-areas have been color-coded. When multiple runs were performed, they were depicted as different rows.

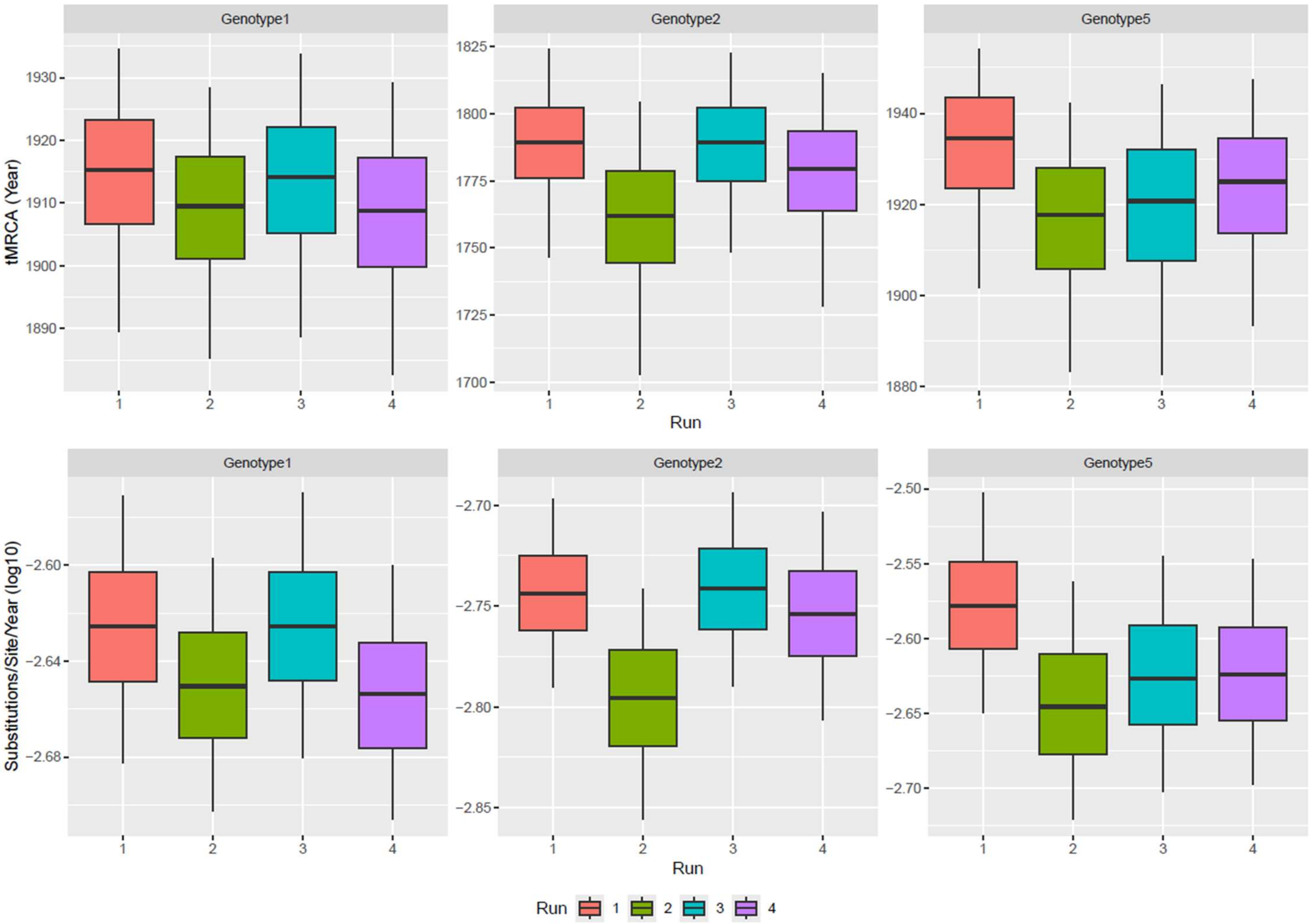

Figure S2. Boxplot depicting the tMRCA posterior probability (upper panel) and mean evolutionary rate (expressed in base-10 logarithm) (lower panel). Graphs are depicted for the genotype for which multiple runs were performed.

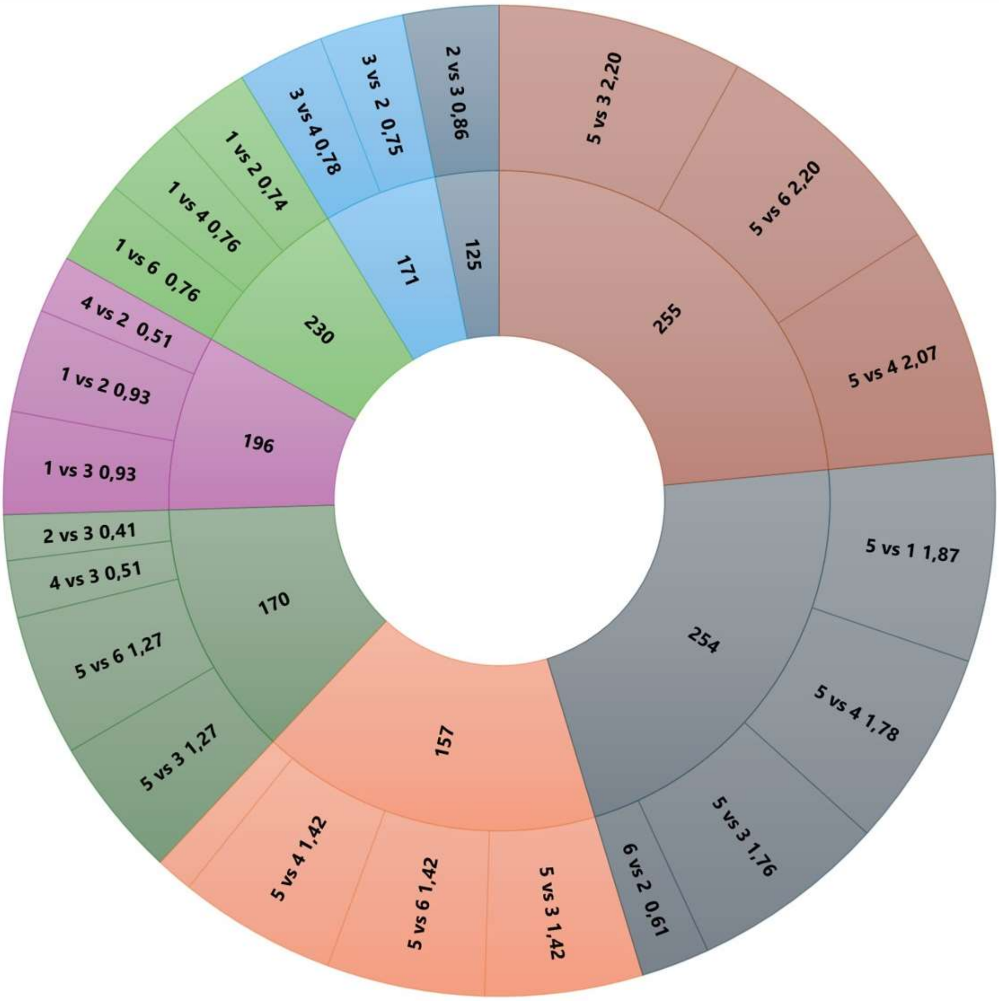

Figure S3. Graphical summary of the amino acid positions where a differential diversifying selection was detected among genotypes. The inner circle reports the involved position, while, in the outer one, the involved genotype pairs are reported. The size of the slices is proportional to the dN/dS difference (reported within the slice) for each genotype pair.
